# Credibility, educational quality, and specialty-specific depth of meniscal injury information on Douyin: a cross-sectional study

**DOI:** 10.7717/peerj.21471

**Published:** 2026-06-26

**Authors:** Wen Zhang, Chang Hu, Yijie Xi, Lihong Xie

**Affiliations:** 1 School of Physical Education, Jiangxi Normal University, Nanchang, China; 2 Nanfang Hospital, Southern Medical University, Guangzhou, China

**Keywords:** Meniscal injury, Douyin, Chinese, Information quality, Health communication

## Abstract

**Background:**

We evaluate the credibility, overall educational quality, and specialty-specific depth of Douyin videos on meniscal injury, examined the relationship between information quality and dissemination metrics, and proposed improvement strategies.

**Methods:**

On July 31, 2025, two Chinese keywords were searched under three sorting modes; the top 50 results per query were collected (*n* = 300). Videos were included if they substantially addressed meniscal injury, symptoms, diagnosis, treatment, rehabilitation, or care-seeking. After de-duplication and exclusions, 143 videos remained. A three-tier framework was applied using the Journal of the American Medical Association (JAMA) benchmarks, the modified DISCERN tool, Global Quality Score (GQS), and the American Academy of Orthopaedic Surgeons (AAOS) Meniscus-specific Score (MSS). Two trained sports-medicine raters independently and blindly scored all videos with adjudication for disagreements. Kruskal-Wallis tests compared scores across video source and content categories; Spearman correlation assessed associations among tools.

**Results:**

Median (IQR) scores were JAMA 2 (2–2), DISCERN 2 (2–2), GQS 2 (1–3), and MSS 3 (1–8), indicating low transparency, evidence disclosure, and educational value, with variable meniscus-specific depth. Across source categories, JAMA, DISCERN, and MSS differed significantly (all *p* < 0.001), whereas GQS did not. A cross content categories, JAMA (*p* = 0.014) and MSS (*p* = 0.028) differed significantly, whereas DISCERN and GQS did not. Videos by professional physicians and rehabilitation therapists outperformed those by fitness enthusiasts and patients on MSS and some credibility metrics. By content, “treatment pathway and surgical options” achieved higher MSS, whereas “rehabilitation training and postoperative recovery” more often missed key points. Correlations were positive across tools; GQS correlated most strongly with MSS (*r* = 0.638, *p* < 0.01), while JAMA and DISCERN showed moderate correlations with MSS, suggesting complementarity between professional depth and credible disclosure. Among dissemination metrics, only comments differed significantly across video source and content categories (*p* = 0.041; *p* = 0.023); likes, saves, and shares did not consistently reflect quality, indicating a decoupling between engagement and quality.

**Conclusions:**

Using a three-tier framework, we found low professionalism, transparency, and educational value in Douyin videos on meniscal injury. Engagement appears decoupled from quality. We recommends a minimum disclosure checklist, platform-level structured publishing and credibility labels with weighted distribution, and standardized short videos by medical institutions integrated with offline education. The findings provide empirical evidence and practical implications for improving health information quality and informing governance strategies on Chinese short-video platforms.

## Introduction

Meniscal injury is among the most common lesions of the knee (Adams, Houston & Cameron, 2021), involving fissuring, tearing, or degeneration of the fibrocartilage of the medial or lateral meniscus, and directly compromising shock absorption, load distribution, and joint stability (Fox et al., 2015; Ozeki, Koga & Sekiya, 2022). Acute meniscal injuries are commonly associated with torsional or shearing loads on the knee, such as sudden deceleration with pivoting, twisting during squatting, or direct trauma. In contrast, chronic or predisposing risk factors for meniscal pathology include age-related degeneration, obesity, lower-limb malalignment, and muscular weakness or imbalance (Poehling, Ruch & Chabon, 1990; Adams, Houston & Cameron, 2021; Yokoe et al., 2023; Xing, 2023). Clinically, patients may present with pain, clicking, locking, swelling, and restricted range of motion, which in turn reduce work and sports capacity, increase the risk of falls, and may accelerate the progression of knee osteoarthritis, thereby elevating healthcare burden and diminishing quality of life (Bernstein, 2000; Hare et al., 2017; Graham et al., 2024; Chen et al., 2025).

Epidemiological studies report that the mean annual incidence of meniscal injury in the general population is 66–70 per 100,000 people (Mitchell et al., 2016); MRI can detect meniscal abnormalities in about 19–35% of asymptomatic individuals (Englund et al., 2011; Beals et al., 2016; Culvenor et al., 2018). In middle-aged and older adults, with declining meniscal elasticity and accumulating degenerative changes, approximately 30–40% have degenerative lesions (Beaufils et al., 2017a, 2017b; Özdemir & Kavak, 2019); individuals with obesity have a 2 to 3-fold higher risk compared with those of normal weight (Ford et al., 2005; Ang, Wong & Lui, 2022; Deng, 2023). Given the high occurrence of meniscal injury, its marked impact on function and quality of life, and its association with the onset and progression of knee osteoarthritis, prevention, early identification, and standardized management are of significant public health importance.

Most mild-to-moderate meniscal injuries can achieve symptom relief and functional improvement through load reduction, adherence to rehabilitation training, appropriate pharmacotherapy, and correction of poor posture and movement patterns, although a subset of patients still experience recurrent pain, locking, and swelling (Sonare, Deshpande & Rahule, 2015; Thorlund et al., 2018; Duong et al., 2023). While arthroscopic debridement alone may provide short-term relief, it can increase the long-term risk of joint degeneration and impose a greater economic burden (Khan et al., 2014; Monk et al., 2017; O’Connor et al., 2022). Evidence from research and clinical practice indicates that structured rehabilitation, weight management, and optimization of lower-limb loading can significantly reduce symptoms and the risk of reinjury (Kise et al., 2016; Bennell et al., 2021; Logerstedt et al., 2021). For selected tear patterns, timely meniscal repair or reconstruction combined with standardized rehabilitation offers superior long-term joint-preserving benefits compared with debridement alone (Doral et al., 2018; Ronnblad et al., 2020). Therefore, disseminating accurate and actionable meniscus-related knowledge through mainstream communication channels has become a key issue influencing public self-assessment, care-seeking, and rehabilitation decision-making.

Meniscal injury is also frequently observed in younger and physically active individuals, particularly those involved in sports and exercise. As these populations are generally more familiar with digital media and more likely to seek health-related information through online and social media platforms, evaluating the quality of meniscal injury information on short-video platforms is especially relevant. With the widespread adoption of mobile internet and smart devices, short-video platforms are reshaping how the public accesses health information and how patients and clinicians communicate (Huxley et al., 2015; Forgie et al., 2021; Ren, Ghazali & Kamarudin, 2025). Existing studies suggest that social media, leveraging intuitive imagery and concise narratives, can translate complex medical concepts into content that is easier to understand and remember, enabling users to acquire disease knowledge, indications for seeking care, and rehabilitation essentials in a short time *via* keyword search (O’Connor et al., 2021; Lobo et al., 2022; Terry et al., 2023). Against the backdrop of uneven healthcare resource allocation and limited outpatient time, such visual dissemination offers advantages of convenience, low cost, repeatable access, and relative privacy, and is becoming an important channel for supplementing and verifying health information (Moorhead et al., 2013; Gupta, Khan & Kumar, 2020).

In a platformized media ecology, short video platforms are not neutral conduits of information; the visibility and credibility of content are jointly shaped by platform logics, algorithmic curation, and engagement driven amplification. Studies indicate that, due to low production thresholds, heterogeneous sources, and the technical complexity of evidence-based literature, the quality of disease-related information on these platforms is highly variable (Dalmer, 2017; Song et al., 2021; Jain et al., 2024). Research across multiple conditions has shown that short video content commonly exhibits unclear levels of evidence, omission of key risk information, exaggerated claims of efficacy, commercial bias, and incorrect demonstrations of exercises or procedures, which can mislead patient judgment and treatment choices and delay timely care (Ragab et al., 2020; Sun, Zheng & Wu, 2023; He et al., 2025).

Douyin is a leading short-video platform in mainland China, with more than 600 million daily active users and a penetration rate exceeding 87% among mobile internet users (Bondy et al., 2020; Yang et al., 2022). Although Douyin and TikTok share a common corporate origin and similar platform logic, they are distinct applications serving different markets and media ecosystems. Owing to its massive user base, diverse content environment, and advanced video creation tools, Douyin has become an important channel for health information dissemination and public engagement (Cheng et al., 2025). Despite this growing role, little is known about the quality, credibility, and meniscus-specific completeness of meniscal injury information available on Douyin. This gap is important because inaccurate or incomplete short-video content may influence users’ understanding, care-seeking behavior, and rehabilitation decisions, particularly among younger and digitally engaged populations. Therefore, this study focuses on Douyin to provide a multidimensional assessment of meniscal injury-related short videos by evaluating their credibility, overall educational quality, and specialty-specific depth, while also examining how these quality dimensions relate to dissemination characteristics, thereby generating evidence to inform efforts to improve online health communication around meniscal injury.

## Materials and Methods

### Search strategy and data processing

To systematically retrieve Douyin videos related to meniscal injury, we searched using two Chinese keywords: “meniscal injury” (“半月板损伤”), a high-frequency term commonly used by the public, and “knee meniscal injury”(“膝关节半月板损伤”), which emphasizes the anatomical site and is more consistent with clinical terminology. These search terms were selected to maximize topic specificity and relevance to meniscal pathology in the Chinese-language Douyin environment, as both are commonly used in Chinese clinical communication and public health content to refer specifically to meniscal injury. In this study, meniscal injury was defined as traumatic or degenerative pathology of the medial or lateral meniscus; videos involving concomitant knee injuries were included only when meniscal injury was the primary focus. Broader terms such as “knee injury” or “knee cartilage” were not adopted because they would likely retrieve a substantial volume of videos not specifically related to meniscal pathology, thereby reducing the specificity and interpretability of the sample. Douyin provides three sorting options for search results: “comprehensive ranking,” “latest,” and “most liked.” To capture common user habits and information preferences, on July 31, 2025, we searched each keyword under all three sorting modes and collected the top 50 videos per query, yielding 300 initial records. This keyword-based retrieval and ranked-result sampling approach is consistent with prior cross-sectional studies evaluating health information quality on online video and short-video platforms (D’Ambrosi & Hewett, 2024). The “top 50” threshold was chosen for two reasons: (1) Douyin’s search ranking integrates match and relevance, so higher-ranked results better reflect the search topic; and (2) typical users follow a “least effort” pattern and are more likely to browse only the top results, making the top 50 more representative of the practically accessible information environment. The use of a top-ranked sampling threshold is also supported by previous meniscus-related video studies that evaluated the most visible videos returned by platform search systems (Kunze et al., 2020).

**Figure 1:**
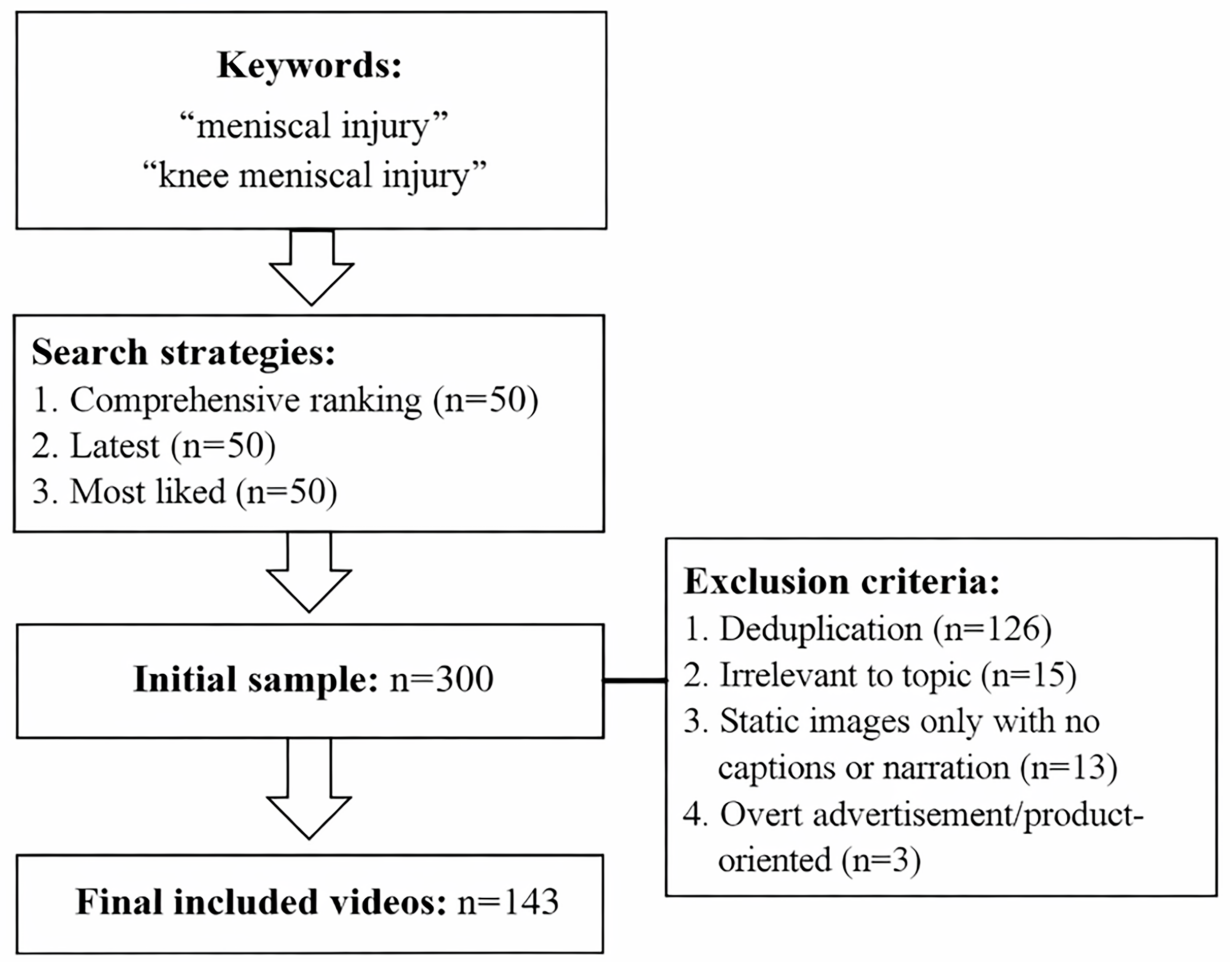
Search strategy and video screening procedure. The study’s search strategy and video screening procedure in a single workflow diagram, illustrating how Douyin videos were identified under predefined keywords and sorting modes, then de-duplicated and excluded according to preset criteria, and finally retained as the analytic sample for subsequent quality assessment.

To minimize the influence of personalization, prior behavior, and location on search outcomes, we created a dedicated Douyin account, disabled personalized recommendations and location permissions, and conducted all searches and data recording on the same device and network. No interactive behaviors (download, like, comment, favorite, or share) were performed during the process. We then deduplicated records using the video ID as a unique identifier (*n* = 126), and excluded videos that were irrelevant to the topic (*n* = 15), consisted only of static images without captions or narration (*n* = 13), or were overtly advertisement/product-oriented (*n* = 3), leaving 143 videos for subsequent analysis (see Fig. 1). Based on a predesigned coding schema, structured data extraction and management were conducted in Microsoft Excel, including the following fields: video URL identifier, upload date, video duration (minutes), number of likes, comments, saves, and shares.

### Assessment of reliability and quality

This study used a three-tier framework to evaluate the information quality and educational value of the included videos, encompassing source transparency and structural credibility, overall educational quality, and meniscus-specific content depth. Informed by previous online health information research (Kunze et al., 2020; Cai et al., 2024a), this framework integrates generic quality assessment tools with a disease-specific content measure to provide a more comprehensive evaluation of video-based educational content.

Source transparency and structural credibility were assessed using the Journal of the American Medical Association (JAMA) benchmark criteria and the modified DISCERN tool. These instruments, together with the Global Quality Score (GQS) and condition-specific content measures, have been widely used in previous studies evaluating online health information and video-based educational materials, supporting their applicability for multidimensional quality assessment in this context. Specifically, the JAMA criteria comprise four independent items (Authorship, Attribution, Currency, and Disclosure), with one point awarded for each item met, yielding a total score of 0–4; higher scores indicate better source transparency and benchmark-based credibility (Ergenç & Uprak, 2023; Kwan et al., 2024). The modified DISCERN tool includes five yes/no items, with “yes” scored as one point, for a total of 1–5; higher scores indicate stronger structural credibility and information quality. The modified DISCERN tool has been widely applied in video-based health information research (Charnock et al., 1999; Rees, Ford & Sheard, 2002; Cai et al., 2024b). Overall educational quality was evaluated using the Global Quality Score (GQS), which provides a holistic judgment of video educational quality across five criteria, with a total score of 1–5; higher scores reflect better overall educational quality, the GQS has been extensively adopted in prior studies of online medical videos (Chen, Pan & Zuo, 2022; Barlas et al., 2023). Meniscus-specific content depth was assessed with the American Academy of Orthopaedic Surgeons (AAOS) Meniscus-specific Score (MSS), which includes 20 items covering five domains: patient presentation and symptoms, meniscal anatomy, diagnosis and assessment, treatment options, and postoperative course and expectations. Each item is scored as present = 1 point, for a total of 0–20; higher scores indicate higher meniscus-specific educational completeness and content coverage. As a condition-specific content checklist derived from previously published meniscus-related video assessment criteria, the MSS was used to improve content relevance and topic-specific interpretability (Kunze et al., 2020). It should be noted that these tools do not directly verify factual accuracy item by item against clinical guidelines or expert consensus statements. Rather, they assess information quality across multiple dimensions and therefore cannot be regarded as direct evidence of misinformation or scientific incorrectness.

The rating team consisted of two researchers with complementary disciplinary backgrounds, reflecting an integrated sports-medicine perspective. One rater was a doctoral candidate in sport sciences with expertise in exercise anatomy and physiology, sports injury and rehabilitation, and sports medicine. The other was a master’s student with a medical background who had received systematic training in evidence-based medicine and clinical research methods. This interdisciplinary configuration was considered appropriate because prior studies have predominantly relied on a single, clinician-centric perspective (Li et al., 2024; Su et al., 2025), whereas meniscal injury is closely related to both clinical care and sport-specific contexts, allowing the assessment process to attend to both medical relevance and practical applicability. Before formal assessment, both raters underwent systematic training in evidence appraisal and critical evaluation and completed standardized training and calibration for the JAMA, GQS, MSS, and modified DISCERN instruments. To promote scoring consistency, the raters jointly reviewed the scoring criteria, discussed item interpretations and decision thresholds, and developed a written coding manual. Relevant clinical references were consulted only to standardize conceptual understanding of meniscal pathology and support consistent application of the instruments, rather than to perform item-by-item factual verification. The raters then conducted pilot assessments on no fewer than 20 preliminary videos, compared scoring discrepancies, and refined the operational rules until a consistent scoring approach was established.

During the formal phase, each video was independently evaluated by both raters under across all four instruments, with each reviewer blinded to the other’s scores. In cases of disagreement, the raters first referred to the coding manual and discussed the case to reach consensus; if disagreement persisted, the video was referred to a third adjudicator. The adjudicator was an associate professor with expertise in exercise human science, anatomy, physiology, and exercise rehabilitation, who had not participated in prior rating or data handling. The adjudicator made an independent judgment based on the coding manual and predefined scoring rules to enhance objectivity and methodological rigor.

### Ethical considerations

This study did not involve clinical data, human participants, biological specimens, or laboratory animals. All materials consisted of publicly accessible Douyin short videos. For this descriptive content analysis study, ethical approval was obtained from the Ethics Committee of the School of Physical Education, Jiangxi Normal University (Decision No.: IRB-JXNU-PEC-20250405, Date: April 5, 2025).

### Statistical analysis

Data were analyzed using SPSS 26.0 (IBM Corp., Armonk, NY, USA). Descriptive statistics were first used to summarize the basic characteristics of the included videos, including video duration, upload year, engagement indicators (likes, comments, saves, and shares), and quality assessment scores (JAMA, modified DISCERN, GQS, and MSS). Normality was assessed for continuous variables. Normally distributed data are presented as mean ± standard deviation (SD), whereas non-normally distributed data are presented as median (M) and interquartile range (IQR), (M (IQR)) (Kim, 2013; Mishra et al., 2019).

To examine whether video dissemination characteristics and quality differed by category, the Kruskal-Wallis test was used to compare video duration, engagement indicators, and quality assessment scores across video source categories and video content categories (Ruxton & Beauchamp, 2008; Ostertagová, Ostertag & Kováč, 2014). Spearman correlation analysis was performed to assess the relationships among JAMA, modified DISCERN, GQS, and MSS scores, thereby exploring the associations among source transparency/structural credibility, overall educational quality, and meniscus-specific content depth (Hauke & Kossowski, 2011; Schober, Boer & Schwarte, 2018). A two-tailed *p* < 0.05 was considered statistically significant.

## Results

### Basic features of video

**Figure 2:**
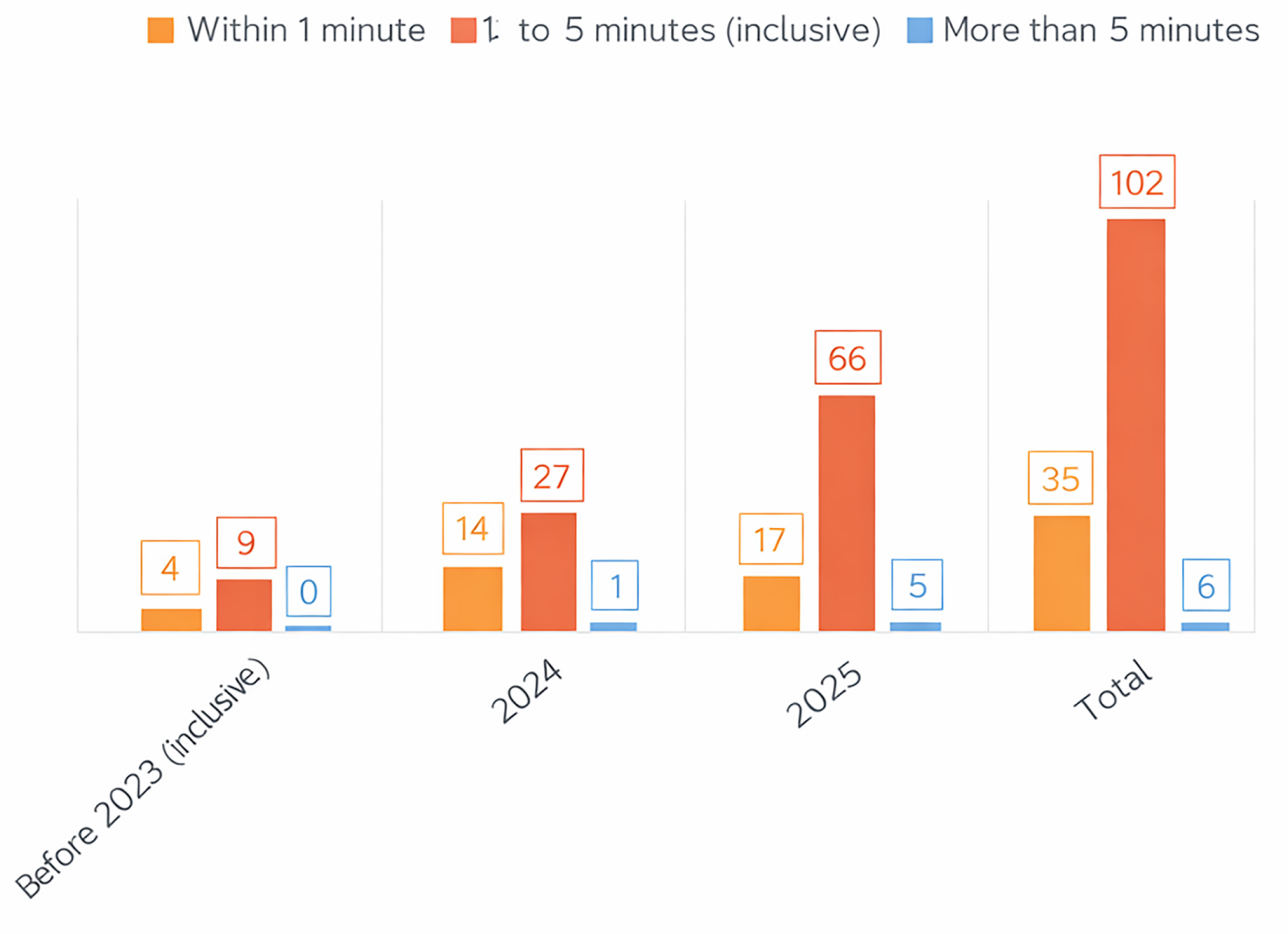
Distribution of video durations by upload year. The durations of the included Douyin meniscal-injury videos are distributed across different upload years.

Building on the preliminary screening, 143 videos related to meniscal injury were included in the final analysis. Of these, 35 (24.48%) were 1 min or shorter, 102 (71.33%) were 1–5 min duration, and six (4.19%) were longer than 5 min. By upload year, 13 videos (9.09%) were published in 2023 or earlier, 42 (29.37%) in 2024, and 88 (61.54%) in 2025 (see Fig. 2).

**Figure 3:**
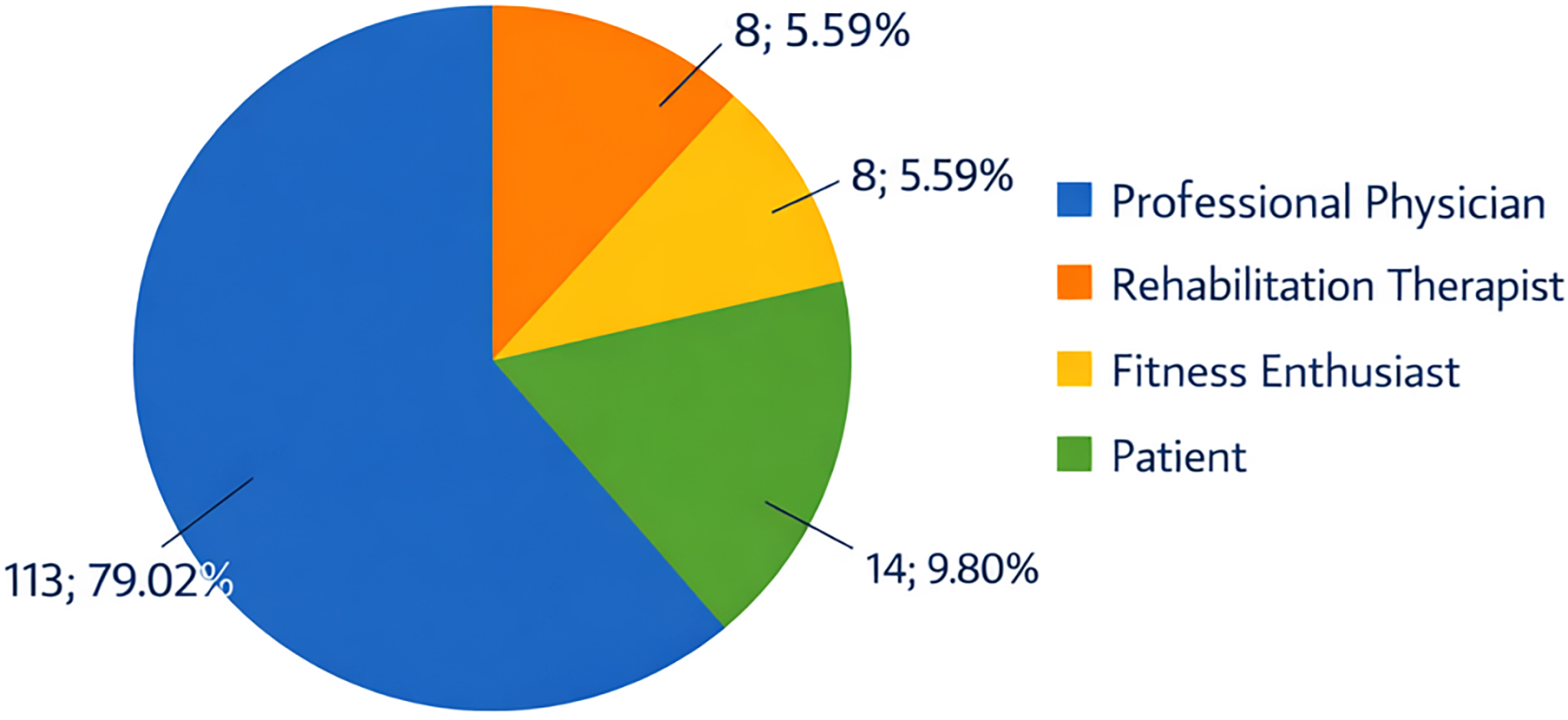
Distribution of video source categories. The final analytic sample of Douyin meniscal injury videos (*n* = 143) is distributed across four source categories.

**Figure 4:**
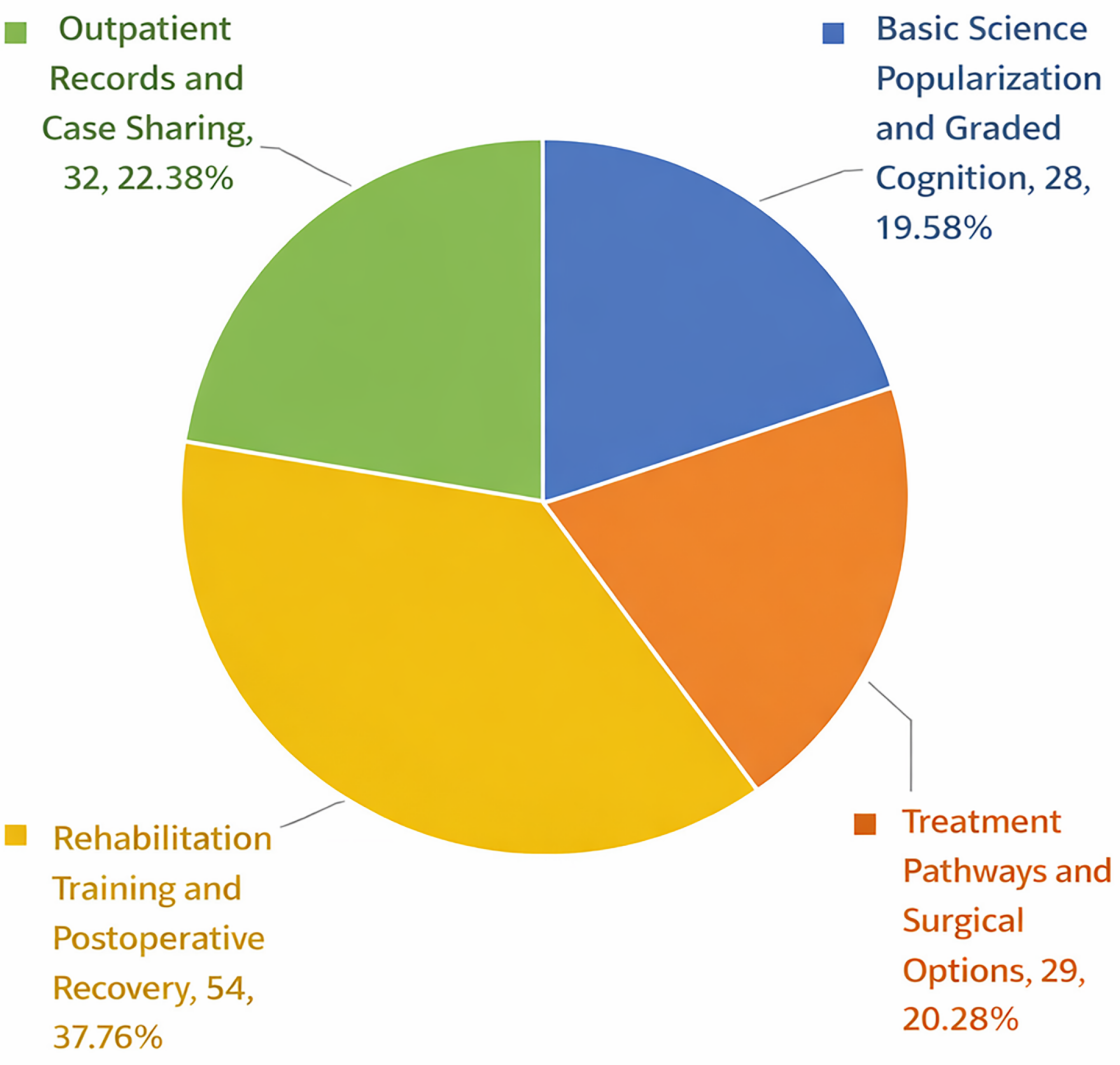
Distribution of video content categories. The final analytic sample is distributed across four video content categories.

To characterize uploader background, videos were classified into four source groups: Professional Physician, Rehabilitation Therapist, Fitness Enthusiast, and Patient. Fitness Enthusiast referred to non-licensed creators whose accounts primarily focused on exercise, gym training, sports performance, or general fitness coaching and who did not present verified medical or rehabilitation credentials. Professional Physician accounted for the largest proportion (113 videos, 79.02%), followed by Patient (14, 9.80%), Rehabilitation Therapist (8, 5.59%), and Fitness Enthusiast (8, 5.59%) (see Fig. 3). To characterize topical focus, videos were further classified into four content categories: Basic Science Popularization and Graded Cognition, Treatment Pathway and Surgical Option, Rehabilitation Training and Postoperative Recovery, and Outpatient Record and Case Sharing. Rehabilitation Training and Postoperative Recovery was the most common category (54 videos, 37.76%), followed by Outpatient Record and Case Sharing (32, 22.38%), Treatment Pathway and Surgical Option (29, 20.28%), and Basic Science Popularization and Graded Cognition (28, 19.58%) (see Fig. 4).

By source category, Professional Physician videos had the highest median likes, saves, and shares, whereas Patient videos had the highest median comments. Rehabilitation Therapist and Patient videos tended to be longer, while Fitness Enthusiast videos were the shortest. Only comments differed significantly across source categories (*p* = 0.041). By content category, Basic Science Popularization and Graded Cognition had the highest median likes, saves, and shares, whereas Outpatient Record and Case Sharing had the highest median comments. Rehabilitation Training and Postoperative Recovery was the most frequent category but had the shortest median duration. Significant differences across content categories were observed for duration (*p* = 0.034) and comments (*p* = 0.023) (see Table 1).

**Table 1:** Sources, content types, and engagement metrics. Descriptive overview of the 143 included Douyin meniscal injury videos, grouping them by publisher source and by content type. For each group, it reports the median (IQR) for video duration and engagement indicators, and it also provides Kruskal-Wallis H statistics and *p* values to test whether these metrics differ significantly across groups.

Category	Video duration M (IQR)	Number of video likes M (IQR)	Number of video comments M (IQR)	Number of video saves M (IQR)	Number of video shares M (IQR)
Video source					
Professional physician	1.38 (1.01, 2.50)	3,731 (1,545, 9,730)	341 (104, 730)	1,941 (876, 6,035)	1,149 (415, 3,615)
Rehabilitation therapist	2.26 (0.78, 2.79)	1,725 (804, 3,845)	113 (54.50, 146)	754.50 (482.50, 3,690)	377.50 (215.50, 692)
Fitness enthusiast	1.22 (0.38, 2.17)	2,925 (2,016.50, 6,846.50)	157.50 (108, 653)	1,739.50 (1,181, 4,535)	818 (547.50, 1,180)
Patient	2.09 (1.10, 3.15)	1,716.50 (1,320, 7,219)	642.50 (476, 1,159)	930.50 (510, 6,614)	572.50 (308, 1,838)
H	2.611	3.296	8.239	2.207	6.229
*p*-value	0.456	0.348	0.041	0.531	0.101
Video content					
Basic science popularization and graded cognition	1.50 (0.55, 3.13)	4,851.50 (2,092.50, 7,789)	398.50 (242, 937)	2,262 (1,042.50, 4,859)	1,163 (700.50, 2,755)
Treatment pathway and surgical option	2.02 (1.21, 2.38)	3,312 (1,654, 9,652)	266 (81, 583)	1,720 (729, 5,125)	804 (260, 2,507)
Rehabilitation training and postoperative recovery	1.22 (0.50, 2.16)	3,109.50 (1,320, 9,516)	194.50 (84, 631)	2,142 (877, 9,186)	818 (356, 3,257)
Outpatient record and case sharing	1.44 (1.10, 3.24)	2,832 (1,048, 9,121.50)	655 (188.50, 1,191)	1,045.50 (292, 3,722.50)	782.50 (244.50, 3,489)
H	8.657	1.121	9.531	5.162	1.937
*p*-value	0.034	0.772	0.023	0.160	0.596

**Note:**

Values are presented as median (IQR); H denotes the Kruskal–Wallis H statistic.

### Assessment of video reliability and quality

Based on median (IQR) results and Kruskal-Wallis H tests, among the 143 included videos, the JAMA and DISCERN scores were both 2, GQS was 2 (IQR 1–3), and MSS was 3 (IQR 1–8), indicating moderate baseline credibility and general quality, with a relatively dispersed distribution for medical specificity (MSS). Between-group analyses showed significant differences by both video source and video content for JAMA (*p* < 0.001; *p* = 0.014) and MSS (*p* < 0.001; *p* = 0.028). There were also significant differences in DISCERN by video source (*p* < 0.001). No significant differences were observed for GQS by either video source or content (see Table 2).

**Table 2:** Analysis of the inter-group differences in video sources and video content. Overall and subgroup distributions of video quality scores for the 143 Douyin meniscal-injury videos, presenting median (IQR) values for JAMA, modified DISCERN, GQS, and the AAOS Meniscus-specific score (MSS). It stratifies results by video publisher source and by video content type. Also provides Kruskal–Wallis H tests and *p* values to identify whether score differences across sources or content categories are statistically significant.

Variable	JAMA M (IQR)	DISCERN M (IQR)	GQS M (IQR)	MSS M (IQR)
Total	2 (2, 2)	2 (2, 2)	2 (1, 3)	3 (1, 8)
**Video source**				
Professional physician	2 (2, 2)	2 (2, 2)	2 (1, 3)	4 (2, 9)
Rehabilitation therapist	2 (1, 2.50)	2 (2, 2)	2.50 (2, 3)	5 (3.50, 6.50)
Fitness enthusiast	1 (0, 1.50)	1 (1, 1.50)	1 (1, 2)	0.50 (0, 1)
Patient	0 (0, 2)	1 (0, 2)	2 (1, 3)	1 (0, 1)
H	30.083	27.846	7.463	36.349
*p*-value	<0.001	<0.001	0.059	<0.001
**Video content**				
Basic science popularization and graded cognition	2 (2, 2)	2 (2, 2)	2 (1, 3)	3 (2, 6.50)
Treatment pathway and surgical option	2 (2, 2)	2 (2, 2)	2 (2, 3)	7 (3, 10)
Rehabilitation training and postoperative recovery	2 (1, 2)	2 (1, 2)	2 (1, 3)	2 (1, 6)
Outpatient record and case sharing	2 (2, 2)	2 (1, 2)	2 (1, 2.50)	4 (1.50, 9)
H	10.631	4.851	3.804	9.125
*p*-value	0.014	0.183	0.283	0.028

**Note:**

Values are presented as median (IQR); H denotes the Kruskal–Wallis H statistic.

### Analysis of the correlation of the assessment tools

Spearman correlation analysis demonstrated associations among all assessment metrics, with varying strengths. Specifically, JAMA was positively correlated with DISCERN (*r* = 0.261, *p* < 0.01), GQS (*r* = 0.213, *p* < 0.05), and MSS (*r* = 0.375, *p* < 0.01). DISCERN was also positively correlated with GQS (*r* = 0.407, *p* < 0.01) and MSS (*r* = 0.374, *p* < 0.01). Among these associations, the strongest correlation was observed between GQS and MSS (*r* = 0.638, *p* < 0.01) (see Table 3).

**Table 3:** Correlation matrix among JAMA, DISCERN, GQS, and MSS. Spearman correlation matrix summarizing the relationships among the four evaluation metrics used in this study—JAMA, modified DISCERN, global quality score (GQS), and the AAOS Meniscus-specific score (MSS). Reports the pairwise correlation coefficients and marks statistical significance at *p* < 0.05 and *p* < 0.01, showing overall positive associations, with the strongest correlation observed between GQS and MSS.

Variable	JAMA	DISCERN	GQS	MSS
JAMA	1			
DISCERN	0.261**	1		
GQS	0.213*	0.407**	1	
MSS	0.375**	0.374**	0.638**	1

**Notes:**

* *p*-value < 0.05.

** *p*-value < 0.01.

## Discussion

Using Douyin as the medium, this study systematically evaluated the credibility (JAMA, DISCERN), overall educational quality (GQS), and specialty-specific depth (MSS) of Chinese-language short videos on meniscal injury. The results showed median scores of 2 for JAMA and DISCERN, 2 (IQR 1–3) for GQS, and 3 (IQR 1–8) for MSS, indicating generally low transparency, evidence disclosure, and overall educational quality, with substantial variability in specialty depth of key meniscal content coverage. Subgroup analyses further indicated that videos posted by Professional Physicians and Rehabilitation Therapists outperformed those by Fitness Enthusiasts and Patients in MSS and some credibility metrics; by content type, videos on Treatment Pathway and Surgical Options had higher MSS, whereas Rehabilitation Training and Postoperative Recovery videos were more prone to missing key points. These findings align with prior studies on TikTok (Sun, Zheng & Wu, 2023), cross-platform comparisons between TikTok and Kwai (Zhao et al., 2024), and multi-platform analyses involving TikTok, WeChat, and Xiaohongshu (Guan et al., 2024). Notably, professional status does not automatically equate to high-quality presentation; structured disclosure and citation of evidence are crucial for quality improvement.

The study also found the strongest correlation between GQS and MSS, indicating that audiences’ subjective evaluations of “overall quality” are highly coupled with the content’s “professionalism.” However, JAMA and DISCERN were only moderately correlated with MSS, suggesting that “professional depth” and “credible disclosure” are complementary dimensions: the former emphasizes the comprehensiveness and usability of the information content, while the latter emphasizes whether the information source is reliable, whether updates are timely, and whether interests are disclosed. Therefore, improving video quality cannot rely solely on adding professional key points; transparency elements such as author credentials and references must also be strengthened. Moreover, considering platform dissemination performance, significant differences by video source and content type were observed only for the “comments” metric, while likes, saves, and shares did not consistently distinguish information quality. This suggests that engagement signals are not quality signals, and platforms are more likely to amplify controversial and emotionally charged topics (Brady et al., 2017; Cuan-Baltazar et al., 2020). This “decoupling of quality and engagement” aligns with prior research, where a preference for instant engagement may weaken quality-based ranking (Madathil et al., 2015; Li et al., 2020; Song et al., 2021).

From the perspectives of content supply and audience demand, the above findings can be better understood in light of previous literature on online health communication. On the supply side, short-video creators often adapt medical information to the platform logic of brevity, visibility, and rapid engagement, which may encourage simplified narratives, action-oriented demonstrations, and selective presentation of benefits over risks (Madathil et al., 2015; Song et al., 2021; Jain et al., 2024). Prior studies across different health topics have shown that social media videos frequently omit evidence levels, contraindications, and uncertainty while emphasizing practicality and immediacy, especially in content related to self-management or exercise instruction (Springer et al., 2020; Sun, Zheng & Wu, 2023; He et al., 2025). Our findings extend this pattern to meniscal injury-related Douyin videos: although rehabilitation training and postoperative recovery was the most common content category, its relatively low MSS suggests that highly actionable content may still lack specialty-specific completeness. In other words, content that is easy to imitate is not necessarily content that is clinically adequate.

On the demand side, previous studies suggest that patients with musculoskeletal complaints often seek clear, rapid, and practical guidance, particularly when they hope to relieve pain quickly or return to sport and daily activity as soon as possible (Pihl et al., 2016; Ahmed et al., 2022). Social media research has further shown that users tend to prefer concise, emotionally engaging, and visually intuitive content, which can make simplified health messages more competitive in platform environments (Brady et al., 2017; Terry et al., 2023). In this context, the relatively weak relationship observed in our study between engagement indicators and information quality is especially noteworthy. It suggests that the popularity of a video may be driven more by immediacy, relatability, or controversy than by transparency or educational value. Taken together, these findings support the interpretation that the interaction among audience preferences, creator strategies, and platform algorithms may facilitate the circulation of “actionable but incomplete” meniscal injury information, thereby potentially shaping care-seeking and rehabilitation decisions in ways that are not always evidence-based.

Based on these findings, our recommendations align with broader literature on digital health communication and misinformation governance. Previous studies suggest that improving online health information quality requires not only better content from individual creators, but also platform-level interventions and stronger involvement of professional institutions (Moorhead et al., 2013; O’Connor et al., 2021). First, for content creators, establish a “minimum disclosure checklist” for health-related videos, including author and institutional information, references and links, update date, conflicts of interest, indications and contraindications, risks and uncertainties, indications for seeking medical care, and negative recommendations on “what not to do.” For rehabilitation exercise content, clearly define training phases, load restrictions, and safety warnings for common errors. Second, for platform management, provide structured publishing templates and credibility badges on the creation side. On the distribution side, incorporate author credential verification, reference links, conflict-of-interest disclosures, and update dates into ranking as credibility weights, and set sensitive keywords and safety interstitial warnings for high-risk actions to mitigate the decoupling between quality and engagement. Third, for medical institutions and audiences, we recommend that medical institutions produce standardized short videos paired with outpatient education one-pagers, forming a continuous offline-to-online educational pathway *via* clinic QR codes, follow-up text messages, and integrated in-hospital social media. Patients should be encouraged to combine online information with in-person care and avoid self-diagnosis or self-training based solely on short videos.

A comprehensive analysis shows that our findings align with prior research on musculoskeletal conditions (*e.g*., anterior cruciate ligament (ACL), rotator cuff, spine disorders) across YouTube/Douyin and Chinese short-video platforms (Cassidy et al., 2018; Springer et al., 2020; D’Ambrosi et al., 2024; Zhang et al., 2025): overall quality is low; professionals outperform non-professionals yet still lack adequate disclosure and citation; rehabilitation demonstration content often omits risk warnings; and engagement metrics correlate weakly with quality scores. This consistency supports the external validity of our study and suggests the improvement pathway is broadly applicable. Overall, low transparency and incomplete coverage of specialty key points are not isolated cases but common phenomena in short-form health information. Future efforts should coordinate among creators, platforms, and medical institutions to advance structured disclosure, credibility labeling, and standardized, staged rehabilitation information. By linking with real-world data (*e.g*., outpatient records, rehabilitation adherence, functional scales), we can assess the real impact of quality improvements on care-seeking behaviors and rehabilitation outcomes.

## Strengths and limitations

This study has several strengths. First, in terms of methodological design, we pre-specified keywords, sorting methods, and inclusion/exclusion criteria, and used a single account within a consistent search environment to minimize the influence of personalized recommendations, ensuring a transparent and reproducible process. Second, we developed a multidimensional evaluation framework covering general credibility and compliance, overall educational quality, and specialty-specific depth. To assess these dimensions, we applied four complementary tools (JAMA, DISCERN, GQS, and MSS) and reported their results separately, which allowed us to evaluate video quality from multiple perspectives, including credibility, educational value, and specialty-specific depth. Third, the scoring procedure involved standardized training and calibration, independent double-masked assessment by two raters, and third-party adjudication, which helped reduce individual judgment bias and improve the consistency of the evaluation process. Fourth, he sampling strategy was designed to approximate real-world user behavior by using commonly used search terms, relevant sorting modes, and a focus on highly visible videos, thereby reflecting the information environment most likely to be encountered by the general public on the platform. Fifth, the study linked quality assessment with dissemination characteristics, leveraging likes, comments, saves, and shares to explore the relationship between information quality and diffusion performance, helping to illuminate underlying mechanisms of content spread. Finally, by focusing on the Douyin platform in a Chinese context, the study generated evidence closely aligned with the local media environment, providing an empirical basis for targeted content standards and governance strategies.

This study has several limitations. First, data were drawn only from Douyin and captured at a single time point, which may not reflect temporal changes in platform content and limits the generalizability of the findings to other time periods or platforms. Second, although the search strategy was designed to reflect real-world user behavior and was informed by previous platform-based studies, it relied on two predefined keywords and the top-ranked results under each search and sorting condition. As a result, some relevant videos retrievable through alternative search expressions or located beyond the top results may not have been captured. Third, the instruments used in this study were designed to evaluate information quality rather than factual accuracy. They rely mainly on visible information and structured presentation, making it difficult to judge evidence strength, factual correctness, and effect sizes. Accordingly, low scores should not be interpreted as direct evidence of misinformation or scientific incorrectness. Fourth, although standardized training, calibration, double-masked assessment, and third-party adjudication were used to improve scoring consistency, formal inter-rater and intra-rater reliability were not assessed. Finally, this study was conducted in a Chinese platform and communication context. While this enhances the contextual relevance of the findings, the results should not be directly generalized to other linguistic, cultural, or platform environments. In addition, as a cross-sectional study, the observed associations do not support causal inference, and the real-world impact of video quality on care-seeking behavior and rehabilitation outcomes could not be directly assessed.

## Conclusions

This study examined Douyin in a Chinese context and developed and applied a multidimensional evaluation framework: “general credibility and compliance-overall educational quality-specialty-specific depth” to systematically assess and compare the information quality and dissemination characteristics of short videos related to meniscal injury. The findings indicate notable deficiencies in professionalism, transparency, and educational value in current content, while suggesting that platform interaction mechanisms may amplify contentious topics. These results provide empirical evidence and practical implications for improving health information quality and may help inform governance strategies on Chinese short-video platforms.

## Supplemental Information

10.7717/peerj.21471/supp-1 Supplemental Information 1 Extracted metadata, engagement metrics, and itemized plus total quality scores for 143 Douyin/TikTok meniscal-injury videos, with each row representing one video.

10.7717/peerj.21471/supp-2 Supplemental Information 2 All variables and coding rules for the study dataset.

10.7717/peerj.21471/supp-3 Supplemental Information 3 Full scoring criteria for all study variables.

## Additional Information and Declarations

### Data Availability

The following information was supplied regarding data availability:

The raw measurements are available in the Supplemental File.
