# Supplementary material for: Credibility, educational quality, and specialty-specific depth of meniscal injury information on Douyin: a cross-sectional study
Source: PeerJ. 2026 Jun 26;14:e21471. doi: 10.7717/peerj.21471 (PMC13312968; doi:10.7717/peerj.21471)
Supplement: Supplemental Information 2 [file peerj-14-21471-s002.docx]

Codebook

- Source of the Video Publisher

1: Professional Physician
2: Rehabilitation Therapist

3: Fitness Enthusiast

4: Patient

- Video Content

1: Basic Science Popularization and Graded Cognition
2: Treatment Pathway and Surgical Option
3: Rehabilitation Training and Postoperative Recovery
4: Outpatient Record and Case Sharing

- Video Upload Time(year)

1: Befpre 2023
2: 2024
3: 2025

- Video Duration(minutes)
  The numbers represent the actual duration of the video
- Number of Video Likes

The numbers represent the actual Video Likes.

- Number of Video Comments

The numbers represent the actual Video Comments.

- Number of Video Saves

The numbers represent the actual Video Saves.

- Number of Video Shares

The numbers represent the actual Video Shares.

- JAMA1

1:Yes

0: No

- JAMA2

1:Yes

0: No

- JAMA3

1:Yes

0: No

- JAMA4

1:Yes

0: No

- DISCERN1

1:Yes

0: No

- DISCERN2

1:Yes

0: No

- DISCERN3

1:Yes

0: No

- DISCERN4

1:Yes

0: No

- DISCERN5

1:Yes

0: No

- MSS1

1:Yes

0: No

- MSS2

1:Yes

0: No

- MSS3

1:Yes

0: No

- MSS4

1:Yes

0: No

- MSS5

1:Yes

0: No

- MSS6

1:Yes

0: No

- MSS7

1:Yes

0: No

MSS8

1:Yes

0: No

- MSS9

1:Yes

0: No

- MSS10

1:Yes

0: No

- MSS11

1:Yes

0: No

- MSS12

1:Yes

0: No

MSS13

1:Yes

0: No

- MSS14

1:Yes

0: No

- MSS15

1:Yes

0: No

- MSS16

1:Yes

0: No

- MSS17

1:Yes

0: No

- MSS18

1:Yes

0: No

- MSS19

1:Yes

0: No

- MSS20

1:Yes

0: No

- JAMA

Represents the sum of items JAMA1 through JAMA4; higher scores indicate greater accuracy and reliability.

- DISCERN

Represents the sum of items DISCERN1 through DISCERN5; higher scores indicate greater accuracy and reliability.

- GQS

1: Very poor

2: Poor

3: Fair

4: Good

5: Excellent

- MSS

Represents the sum of items MSS1 through MSS20; higher scores indicate higher meniscus-specific educational quality
