# Supplementary material for: Credibility, educational quality, and specialty-specific depth of meniscal injury information on Douyin: a cross-sectional study
Source: PeerJ. 2026 Jun 26;14:e21471. doi: 10.7717/peerj.21471 (PMC13312968; doi:10.7717/peerj.21471)
Supplement: Supplemental Information 3 [file peerj-14-21471-s003.docx]

**Source of the Video Publisher**

1: Professional Physician
2: Rehabilitation Therapist

3: Fitness Enthusiast

4: Patient

**Video Content**

1: Basic Science Popularization and Graded Cognition
2: Treatment Pathway and Surgical Option
3: Rehabilitation Training and Postoperative Recovery
4: Outpatient Record and Case Sharing

**Video Upload Time(year)**

1: Befpre 2023
2: 2024
3: 2025

**Video Duration(minutes)**
The numbers represent the actual duration of the video

**Number of Video Likes**

The numbers represent the actual Video Likes.

**Number of Video Comments**

The numbers represent the actual Video Comments.

**Number of Video Saves**

The numbers represent the actual Video Saves.

**Number of Video Shares**

The numbers represent the actual Video Shares.

**JAMA**

JAMA1: Authorship

JAMA2: Attribution

JAMA3: Currency

JAMA4: Disclosure

**Journal of the American Medical Association(JAMA)**

|  | **Item** | **0** | **1** |
| --- | --- | --- | --- |
| JAMA1 | Author and contributor credentials and their affiliations should be provided. | NO | YES |
| JAMA2 | All copyright information should be clearly listed, and references and sources for content should be stated. | NO | YES |
| JAMA3 | The initial date of posted content and dates of subsequent updates to content should be provided. | NO | YES |
| JAMA4 | Conflicts of interest, funding, sponsorship, advertising, support, and video ownership should be fully disclosed. | NO | YES |

**DISCERN**

**5-point DISCERN reliability tool (DISCERN)**

|  | **Item** | **0** | **1** |
| --- | --- | --- | --- |
| DISCERN1 | Are aims clear and achieved? | NO | YES |
| DISCERN2 | Are reliable sources given for the information used? (published articles cited, a specialist’s opinion) | NO | YES |
| DISCERN3 | Is the information presented balanced and unbiased? | NO | YES |
| DISCERN4 | Are additional sources of information listed for patient reference? | NO | YES |
| DISCERN5 | Are areas of uncertainty addressed? | NO | YES |

**GQS:** Provides a holistic judgment of video educational quality across five criteria, with a total score of 1–5.

**Global Quality Score（GQS）**

| **Grade** | **Description** |
| --- | --- |
| 1 | Poor quality, poor flow of the site, most information missing, not at all useful for patients |
| 2 | Generally, poor quality and poor flow; some information is listed, but many important topics are missing, and it is of very limited use to patients |
| 3 | Moderate quality, suboptimal flow, some important information is adequately discussed, but others are poorly discussed, somewhat useful for patients |
| 4 | Good quality and generally good flow, most of the relevant information is listed, but some topics not covered, useful for patients |
| 5 | Excellent quality and excellent flow, very useful for patients |

**MSS**

**Meniscus-specific Score（MSS）**

|  | **Criterion** | **Item** | **0** | **1** |
| --- | --- | --- | --- | --- |
| MSS1 | Patient presentation | Describes symptoms | NO | YES |
| MSS2 |  | Describes the patient population | NO | YES |
| MSS3 | Information about the meniscus | Describes the anatomy and/or function of the meniscus | NO | YES |
| MSS4 |  | Differentiates meniscal cartilage from articular cartilage | NO | YES |
| MSS5 |  | Explains the poor healing potential of the meniscus | NO | YES |
| MSS6 |  | Mentions progression to arthritis or other ligamentous injury after a meniscal tear | NO | YES |
| MSS7 | Diagnosis and evaluation | Mentions physical examination and findings | NO | YES |
| MSS8 |  | Discusses inability for radiographs to evaluate | NO | YES |
| MSS9 |  | Discusses use of MRI as gold-standard diagnostic imaging modality | NO | YES |
| MSS10 |  | Discusses types of tears (radial, bucket handle, degenerative, flap) | NO | YES |
| MSS11 |  | Describes surgical candidates (young adults or patients with symptoms impacting function or quality of life) | NO | YES |
| MSS12 |  | Describes nonsurgical candidates (older adults with few symptoms) | NO | YES |
| MSS13 | Treatment | Mentions conservative treatment | NO | YES |
| MSS14 |  | Mentions diagnostic arthroscopy and other pathologies that may be addressed concomitantly | NO | YES |
| MSS15 |  | Describes partial meniscectomy | NO | YES |
| MSS16 |  | Describes meniscal repair | NO | YES |
| MSS17 | Postoperative course | Describes complications and outcomes | NO | YES |
| MSS18 |  | Mentions weight-bearing restrictions | NO | YES |
| MSS19 |  | Mentions physical therapy | NO | YES |
| MSS20 |  | Outlines return-to-function timeline | NO | YES |

Note:MRI: magnetic resonance imaging
